# Clinical value and indication for the dissection of lymph nodes posterior to the right recurrent laryngeal nerve in papillary thyroid carcinoma

**DOI:** 10.18632/oncotarget.20275

**Published:** 2017-08-16

**Authors:** Ding-Cun Luo, Xiao-Cheng Xu, Jin-Wang Ding, Yu Zhang, You Peng, Gang Pan, Wo Zhang

**Affiliations:** ^1^ Department of Surgical Oncology, Hangzhou First People’s Hospital, Nanjing Medical University, Zhejiang, China; ^2^ Department of Surgery of Thyroid and Breast, Wujiang District of Suzhou First People’s Hospital, Jiangsu, China

**Keywords:** lymph node posterior to right recurrent laryngeal nerve, neck dissection, PTC, risk factors, metastasis

## Abstract

Lymph nodes posterior to the right recurrent laryngeal nerve (LN-prRLN) are common sites of nodal recurrence after the resection of papillary thyroid carcinoma (PTC). However, the indication for LN-prRLN dissection remains debatable. We therefore studied the relationships between LN-prRLN metastasis and the clinicopathological characteristics in 306 patients with right or bilateral PTC who underwent LN-prRLN dissection. We found that LN-prRLN metastasis occurred in 16.67% of PTC and was associated with a number of the clinicopathological features. The receiver-operator characteristic (ROC) analysis showed that the areas under the ROC curves for the prediction of LN-prRLN metastasis by the risk factors age < 35.5 years, right tumor size > 0.85 cm, lymph node (right cervical central VI-1) number > 1.5, metastatic lymph node (right cervical central VI-1) size > 0.45 cm, and lymph node number in the right cervical lateral compartment > 0.5 were 0.601, 0.815, 0.813, 0.725, and 0.743, respectively.

In conclusion, the risk factors for LN-prRLN metastasis in patients suffering right thyroid lobe or bilateral PTC include age ≤ 35.5 years, right tumor size ≥ 0.85 cm, capsular invasion, metastatic lymph node (right cervical central VI-1) number ≥ 2, metastatic lymph node (right cervical central VI-1) size ≥ 0.45 cm, and metastatic lymph node number in the right cervical lateral compartment ≥ 1. In patients whose risk factors can be identified pre-operatively or intraoperatively, the dissection of LN-pr-RLN should be considered during right cervical central compartment dissection.

## INTRODUCTION

In recent years, thyroid cancer has exhibited the rapidest increase in incidence among solid tumors. This increase is mainly due to an increase in papillary thyroid carcinoma (PTC) [1], which has also resulted in a significant increase in surgical treatment for PTC. However, the lack of a standardized and uniform surgical approach may lead to the postoperative relapse of thyroid cancer, which requires at least 1 additional surgery. Furthermore, the reoperation may increase thyroid tumor dedifferentiation as well as the difficulty and risk of the surgical procedure. Particularly, the incomplete resection of the lymph node posterior to the right recurrent laryngeal nerve in the first surgery can make the residual lymph nodes difficult to excise in the second surgery and even render the complete excision impossible. In this situation, patients who should have had a chance to be cured may thus lose the opportunity [2].

The guideline of the American Thyroid Association (ATA) has outlined the anatomic boundaries of the central neck dissection for thyroid cancer to establish uniformity among all surgeons performing this procedure [3]. While the terminology has now be standardized, controversy remains regarding to the surrounding treatment indication for LN-prRLN in PTC and even the names of the dissection procedure. Some researchers have suggested that routine LN-prRLN dissection is not necessary because of the low metastasis rate of LN-prRLN, the relatively high difficulty of dissection, and the high complication rate [4–10]. Therefore, we designed this prospective study to investigate the clinical value and indication for LN-prRLN dissection in PTC.

## RESULTS

### Lymph node dissection and metastasis in the right cervical central compartment (VI)

#### Number of harvested lymph nodes in the right cervical central compartment (VI)

Among the 306 patients with PTC, the median numbers of harvested lymph nodes in the right cervical central subzone VI were 8, 5 in subzone VI-1, and 3 in subzone VI-2. There was a positive association between the total number of subzone VI lymph nodes and the number of subzone VI-1 lymph nodes harvested. (Z = −14.794, *P* = 0.00) (Table 1).

**Table 1:** Number of harvested right cervical central VI lymph nodes

Right cervical	Lymph node numbers	Z*	*P*
VI-1	5 (3, 8)	−*14.794*	***0.000***
VI	8 (6, 12)		

*Mann-Whitney *U*-test.

#### Lymph node metastasis in the right cervical central compartment (VI)

In this study, the metastatic rates of the VI lymph node, the VI-1 lymph node and the VI-2 lymph node (LN-prRLN) were 50.00% (153/306), 48.04% (147/306) and 16.67% (51/306) respectively in the right cervical central compartment. The metastatic rate of both VI-1 and VI-2 lymph nodes was 14.71% (45/306). Among patients with the VI-1 lymph node metastasis, the LN-prRLN metastasis rate was 30.61% (45/147), whereas among patients without the VI-1 lymph node metastasis, the LN-prRLN metastasis rate was much lower, at 3.77% (6/159).

The chance of having a positive lymph node in the right cervical central subzone VI-2 rises from 3.77% (6/159) if subzone VI-1 is negative for metastasis to 30.61% (45/157) if subzone VI-1 is positive.

### Correlation between LN-prRLN metastasis and the clinicopathological characteristics of PTC

A univariate analysis of data from the 306 patients with PTC indicated a significant association (*P* < 0.05) between LN-prRLN metastasis and gender, age, size and number of right thyroid lobe tumor focus/ foci, the presence of capsular invasion, the presence of right cervical central VI-1 lymph node metastasis and their size, and also metastatic lymph node in the right cervical lateral compartment. However, statistically there is no significant association of LN-prRLN metastasis with the tumor-node-metastasis (TNM) stage, the thyroid stimulating hormone (TSH) concentration, previous and concomitant thyroid disorders, BRAF mutation, and contralateral cervical compartment lymph node status (*P* > 0.05) (Table 2).

**Table 2:** Univariate analysis of LN-prRLN metastasis and clinicopathological characteristics of PTC

Clinicopathological	R-VI-2 lymph node		Metastatic rate	χ^2^	*P*
Characteristics	YES	NO			
Gender					
Female	34	204	14.29%	4.371	0.037
Male	17	51	25.00%		
Age (year)					
≤ 30	13	25	34.21%	10.967	0.012
> 30, ≤ 40	13	59	18.06%		
> 40, ≤ 50	13	78	14.29%		
> 50	12	93	11.43%		
R-Tumor size (cm)					
≤ 0.5	4	110	3.51%	48.259	0.000
> 0.5, ≤ 1	14	96	12.73%		
>1	33	49	40.24%		
R-Tumor number					
< 2	45	252	15.15%	13.188	0.000
≥ 2	6	3	66.67%		
Capsular invasion					
no	24	221	9.80%	41.773	0.000
yes	27	34	44.26%		
R-VI-1 lymph node metastasis					
no	6	153	3.77%	39.614	0.000
yes	45	102	30.61%		
R-VI-1 lymph node metastatic					
number					
≤ 1	5	45	10.00%	15.156	0.000
> 1	40	57	41.24%		
≤ 2	15	67	18.29%	13.250	0.000
> 2	30	35	46.15%		
≤ 3	24	75	24.24%	5.791	0.016
> 3	21	27	43.75%		
R-VI-1 lymph node metastatic Size (cm)^a^					
≤ 0.2	16	69	18.82%	31.186	0.000
> 0.2, ≤ 0.5	12	30	28.57%		
> 0.5, ≤ 1	14	2	87.50%		
> 1	3	1	75.00%		
R-lateral lymph node metastasis					
no	23	236	8.88%	73.608	0.000
yes	28	19	59.57%		
R-lateral lymph node metastatic					
number					
≤ 1	5	3	62.50%	-	1.000
> 1	23	16	71.79%		
≤ 2	7	10	41.18%	3.743	0.053
> 2	21	9	70.00%		
≤ 3	12	14	46.15%	4.352	0.037
> 3	16	5	76.19%		
TNM stage					
I + II	30	135	18.18%	0.592	0.442
III + IV	21	120	14.89%		
TSH (mIU/L)					
≤ 2	25	146	14.62%	1.507	0.471
> 2, ≤ 4	20	89	18.35%		
> 4	6	20	23.08%		
Thyroid disorders					
yes	21	123	14.58%	0.850	0.357
no	30	132	18.52%		
BRAF V600E^b^					
wild-type	9	22	29.03%	0.927	0.336
mutant	31	116	21.09%		
L-VI lymph node metastatic^c^					
No	8	47	14.55%	3.112	0.078
yes	11	26	29.73%		
L-lateral lymph node metastatic^d^					
No	0	2	0.00%	-	0.467
Yes	8	6	57.14%		

Abbreviations: R-, right cervical TNM: tumor-node-metastasis TSH: Thyroid-stimulating Hormone

^a^147 patients exhibited R-VI-1 lymph node metastasis

^b^178 samples were subjected to a *BRAF* V600E test

^c^92 patients with L-VI were resected

^d^16 cases were resected.

The statistically significant parameters derived from univariate variate analysis were subjected to further multivariate logistic regression analysis to identify independent associate variables. After multivariate logistic regression analysis, the statistical association (*P* < 0.05) was maintained for the size of the right thyroid lobe tumor, the presence of capsular invasion, right cervical central VI-1 lymph node metastasis, and right lateral compartment lymph node metastasis (Tables 3 and 4).

**Table 3:** Multivariate analysis assignment list

code	Clinicopathological characteristics	Assignment
X1	Gender	0 for female, 1 for male
X2	Age	0 for ≤ 30 years, 1 for > 30 but ≤ 40 years, 2 for > 40 but ≤ 50 years and 3 for > 50 years
X3	Right tumor size	0 for ≤ 0.5 cm, 1 for > 0.5 but ≤ 1 cm, 2 for > 1 cm
X4	Right tumor	0 for ≤ 3, and 1 for > 3
	number	
X5	Capsular invasion	0 for yes, 1 for no
X6	R-VI-1 lymph node	0 for no, 1 for yes
	metastasis	
X7	R-lateral lymph node	0 for no, 1 for yes
	metastasis	
Y	R-VI-2 lymph node	0 for no, 1 for yes
	metastasis	

**Table 4:** Multivariate logistic regression analysis of LN-prRLN metastasis and clinicopathological characteristics of PTC

High-risk factor	β	S.E.	Wals	*P*	Exp	OR 95% C.I.	
Right tumor size	0.855	0.291	8.631	*0.003*	2.352	1.329	4.160
Capsular invasion	1.107	0.430	6.629	*0.010*	3.026	1.303	7.029
R-VI-1 lymph node metastasis	1.844	0.505	13.345	*0.000*	6.323	2.351	17.009
R-lateral lymph node metastasis	1.387	0.434	10.197	*0.001*	4.005	1.709	9.384

### The predictive value of high-risk clinicopathological factors for LN-prRLN metastasis

The continuous variables (age, tumor size, the number and size of the right cervical central VI-1 metastatic lymph node, and the number of metastatic lymph nodes in the right cervical lateral compartment) that were robustly associated with LN-prRLN metastasis were used to generate ROC curves. The areas under the ROC curve for the prediction of LN-prRLN metastasis by age < 35.5 years, the size of right thyroid lobe tumor > 0.85 cm, the number of right cervical central VI-1 lymph nodes > 1.5, the size of right cervical central VI-1 metastatic lymph node > 0.45 cm, and the number of metastatic lymph nodes in the right lateral compartment > 0.5 were 0.601, 0.815, 0.813, 0.725, and 0.743, respectively (Table 5).

**Table 5:** The predictive value of high-risk clinicopathological factors for LN-prRLN metastasis

High-risk factors	AUC	AUC 95% C.I.		*P*	Max Youden index		
					Cut-off	Sensitivity	Specificity
Age	0.601	0.508	0.695	*0.022*	35.5 years	78.82%	41.18%
Right tumor size	0.815	0.752	0.878	*0.000*	0.85 cm	78.43%	72.40%
R-VI-1 lymph node metastatic number	0.813	0.748	0.879	*0.000*	1.5	78.43%	76.65%
R-VI-1 lymph node metastatic size (cm)	0.725	0.628	0.822	*0.000*	0.45 cm	48.89%	90.20%
R-lateral lymph node metastatic number	0.743	0.655	0.831	*0.000*	0.5	54.90%	92.55%

### Follow up of LN-prRLN dissection for complications

In the present study, the duration of follow-up of all patients ranges from 20 months to 38 months. All patients have their blood parathyroid hormone and calcium levels assayed to assess the post-operative parathyroid function. There was no permanent hypoparathyroidism, which was defined as persistently low blood calcium levels (< 2 mmol/L) and subnormal parathyroid hormone levels by 6 months after surgery. The presence of RLN injury was determined by reviewing the surgical records and postoperative laryngoscopy reports. Four patients developed transient recurrent laryngeal nerve (RLN) paralyses and recovered within 2 months of surgery, 2 patients exhibited pleura rupture (which was recognized during surgery and repaired), and 5 patients developed chylous leak which resolved within 2 weeks of surgery. Horner’s syndrome was not observed in this study, and none of the patients developed recurrence.

## DISCUSSION

Approximately 30% to 80% of patients with PTC present with cervical lymph node metastases at the time of diagnosis, and the cervical lymph node metastasis rate in microcarcinoma is as high as 47.6% [11–12]. The LN-prRLN is not uncommon sites of nodal recurrence in PTC [5, 10, 13]. In this study, we found 16.67% of PTC with the LN-prRLN metastasis, which falls into the range of the metastatic rate (11.38%–26.67%) reported previously [5–8]. To this end, we have recruited 306 PTC cases to identify the potential factors that can be used as the indication for LN-prRLN dissection. Specifically, a univariate analysis followed by the multivariate logistic regression analysis indicated a significant correlation between LN-prRLN metastasis and several risk factors.

### LN-prRLN metastasis strongly correlates with tumor size

In this study, the size of the right tumor was established to be an independent risk factor for LN-prRLN metastasis. We categorised the right tumor lesion into 3 groups: diameter ≤ 0.5 cm, > 0.5–1 cm, and > 1 cm. The LN-prRLN metastasis rates in these groups were 3.51%, 12.73%, and 40.24%, respectively. These rates significantly differed between the diameter ≤ 0.5 cm group and the diameter > 0.5–1 cm group (*P* < 0.01) and between the diameter > 0.5–1 cm group and the > 1 cm group (*P* < 0.01) (Table 2). Thus, the tumor diameter was positively correlated with the LN-prRLN metastasis rate. The larger the tumor, the higher the metastatic rate. In patients with a tumor diameter ≤ 0.5 cm, lymph node dissection is not necessary because the tumor metastasis rate of this group was very low. Based on the ROC curve analysis, a tumor size > 0.85 cm has a reasonable sensitivity of 78.43% and specificity of 72.40% in predicting LN-prRLN metastasis. Therefore, we recommend that a tumor diameter ≥ 0.85 cm is an indicator for LN-prRLN dissection.

### Positive correlation between the degree of right cervical central VI-1 lymph node metastasis and LN-prRLN metastasis

In this study, the median number of harvested lymph nodes in the right cervical central was 5 for the VI-1 dissection, and 3 for the VI-2 dissection. Notably, the rate of lymph node metastasis differs between these 2 subzones. It has been reported to be 36.59–49.05% in the right cervical central VI-1 subzone and 5.76–26.67% in the right cervical central VI-2 subzone [4–10]. The current study showed that the lymph node metastasis rate in the right cervical central VI-2 subzone was 16.67%. Considering the 36.59–49.05% metastasis rate in the right cervical central VI-1 subzone, VI-1 subzone dissection, which is relatively straightforward and low risk, is a generally acceptable procedure.

The consideration is different for right cervical central VI-2 subzone dissection given a relatively lower rate of metastasis and technically more difficult and risky surgery. Nevertheless, our study has demonstrated that the probability of the LN-prRLN metastasis was 30.61% (45/147) in patients with right cervical central VI-1 lymph node metastasis, whereas this probability was only 3.77% (6/159) in patients without right cervical central VI-1 lymph node metastasis. Furthermore, the number of metastatic lymph nodes in the right cervical central VI-1 subzone was found to be positively correlated with the risk of LN-prRLN metastasis. The ROC curve analysis identified > 1.5 metastatic lymph nodes as a cut-off with reasonable sensitivity of 78.43% and specificity of 76.65%. Based on this finding, we recommend that ≥ 2 metastatic lymph nodes in the right cervical central VI-1 subzone are an indicator for LN-prRLN dissection.

Based on the size of metastatic lymph nodes in the right cervical central VI-1 subzone, we conducted a stratified analysis that revealed significant differences (*P* = 0.000) among the groups with metastatic lesions ≤ 0.2 cm, > 0.2 cm–0.5 cm, > 0.5 cm–1 cm, and >1 cm. For metastatic lymph lesions in the right cervical central VI-1 subzone ≤ 0.2 cm, > 0.2 cm–0.5 cm, and > 0.5 cm–1 cm, the LN-prRLN metastasis rates were 18.82%, 28.57%, and 87.50%, respectively. These results suggested a positive correlation between the size of metastatic lymph nodes in the right cervical central VI-1 subzone and the risk of LN-prRLN metastasis. Moreover, the ROC curve analysis identified metastatic lymph nodes > 0.45 cm as an intervention threshold (sensitivity of 48.89% and specificity of 90.2%). We conclude that metastatic lymph nodes in the right cervical central VI-1 subzone ≥ 0.45 cm are an indicator for LN-prRLN dissection.

### Lymph node metastasis in the right cervical lateral compartment is a high-risk factor for LN-prRLN metastasis

In this study, 59.57% patients with metastatic lymph nodes in the right cervical lateral compartment harbored LN-prRLN metastasis, compared to 8.88% of those patients without. The difference is highly significant (*P* = 0.000), and the ROC curve analysis identified > 0.5 metastatic lymph nodes in the right cervical lateral compartment as a diagnostic threshold (sensitivity of 54.90% and specificity of 92.55%). We recommend that the metastatic lymph node in the right cervical lateral compartment is an indicator for LN-prRLN dissection.

### Age and LN-prRLN metastasis

In this study, the average patient age was 45.10±11.87 years (range 8 to 74 years). We divided patients into 4 groups according to age: ≤ 30 years, > 30 years–40 years, > 40 years–50 years, and > 50 years. We found that the LN-prRLN metastasis rates in these 4 groups were 34.21%, 18.06%, 14.29%, and 11.43%, respectively, and these rates significantly differed (*P* < 0.05). The rate of LN-prRLN metastasis appeared to be inversely correlated with age, suggesting that younger patients are prone to LN-prRLN metastasis. Specifically, the ROC curve analysis indicated that age < 35.5 years (sensitivity of 78.82% and specificity of 41.18%) was a reasonable cut off for possible LN-prRLN metastasis. This finding is consistent with the data reported by Zhang et al. [8]. Based on above findings, we suggest that age ≤ 35.5 years may be an indicator for LN-prRLN dissection.

### Correlation between LN-prRLN metastasis and capsular invasion, gender, and cancer lesion number

While patients’ gender, the presence of capsular invasion, and the number of cancer lesions were shown to be significant on univariate analysis, only the capsular invasion remains significant on multivariate logistic regression analysis. This disparity may be due to the relatively small sample size. In clinical practice, the capsular invasion is an indicator for LN-prRLN dissection. LN-prRLN dissection can still be performed in male patients with multifocal thyroid carcinoma but the value is less certain.

### LN-prRLN metastasis is not associated with TSH concentration, TNM stage, previous concomitant thyroid disorders, BRAF mutation, or lymph node metastasis in the left cervical compartment

A comprehensive analysis of TSH concentration, TNM stage, previous concomitant thyroid disorders, and lymph node metastasis in the left cervical compartment was conducted in all 306 patients enrolled in this study. The statistical analysis did not identify correlations between any of these factors and LN-prRLN metastasis (*P* > 0.05). In this study, the rate of BRAF V600 mutation was 82.58% (147/178), which was consistent with the 29–83% mutation rate reported in the literature [14]. Li et al reported that BRAF mutations in PTC may predict cervical lymph node metastasis [15]. However, our study showed that the mutation rate was 77.50% (31/40) in patients with LN-prRLN metastasis and 84.06% (116/138) in patients without LN-prRLN metastasis, and these rates did not significantly differ (*P* = 0.336). The disparity between our study and Li’s may be due to the relatively small sample size in our study.

### The range of LN-prRLN dissection should be defined, and LN-prRLN dissection is safe and feasible

At present, the boundaries of LN-prRLN dissection has not been clearly defined. According to this study and our clinical experience, we define the area posterior to the right RLN segment between laryngeal entry point and the intersection of the RLN with the innominate artery as the range of LN-prRLN dissection. Some researchers do not include the area above the level of the inferior thyroid artery in their LN-prRLN dissection to avoid injury of the right superior parathyroid and the associate blood supply [2]. However, this region usually includes some lymph nodes. If the nodal relapse does occur in these left-behind lymph nodes, it will be even more difficult to preserve the parathyroid gland and its blood supply in revision surgery. Actually, the parathyroid and its vessels can be protected from surgical injury if a careful and fine dissection is performed in the primary dissection. The right lower parathyroid and its vessels can be preserved at the anterior-lateral sheath of the common carotid artery during right cervical central VI-1 dissection and should not be easily injured in the right cervical central VI-2 dissection. The intersection between the innominate artery and the tracheoesophageal groove, which is close to the right lung apex and apical pleura, is defined as the inferior boundary of the right cervical central VI-2 subzone. Some black and dust-catching lymph nodes usually present in this area and downward toward the mediastinum. Therefore, the dissection that reaches the level of the innominate artery or the right apical pleura is sufficient. Adipose tissue dissection in the right cervical central VI-2 subzone should be carefully performed in anatomic layers while protecting the trachea and esophagus, and the prevertebral fascia should be kept intact. Traction and electrical cautery burns on the RLN should be avoided, and the capillaries on the surface of the RLN should be preserved. Complete lymphatic and adipose tissue dissection can result in a variety of complications such as RLN injury, pleura rupture, chylothorax, and Horner syndrome, because of the deep and narrow working space in the right cervical central VI-2 subzone. Therefore, an experienced surgeon is required for this thyroid surgery.

In the present study, no patient suffered permanent hypoparathyroidism or Horner’s syndrome following surgery, but 1.3%, 0.7%, and 1.6% of patients developed RLN injury, pleura rupture, and chylous leak, respectively. Overall, the rate of surgical complications was very low in this study. Pleura rupture was identified during surgery and repaired immediately, and patients with chylous leak and transient RLN injury recovered within 2 - 4 weeks of surgery. Therefore, we conclude that LN-prRLN dissection is safe and feasible under experienced hands.

## MATERIALS AND METHODS

### General clinical materials

A prospective study was designed as follows: 306 consecutive PTC patients (right PTC/ bilateral PTC) underwent LN-prRLN dissection conducted by a surgical team over a period of 18 months (from March 2014 to September 2015). Patients who had a history of other malignant tumors were excluded.

Among these patients, 68 were males and 238 females. 207 patients were with right PTC and 99 patients with bilateral PTC. 187 patients were diagnosed with a single lesion, and 119 with multiple lesions. A total of 224 tumors were classified as micro-tumors (diameter ≤ 1 cm) according to the WHO criteria. Furthermore, 51 patients had positive LN-prRLNs, and 255 patients had negative LN-prRLNs. Four patients presented with hyperthyroidism, 10 patients exhibited subclinical hypothyroidism, 82 patients had nodular goiters, 44 patients suffered from chronic thyroiditis, 43 patients were diagnosed with Hashimoto’s disease, and 13 patients exhibited adenoma. The study was approved by the Ethics Committees of Hangzhou First People’s Hospital, and all participants provided informed consent.

### LN-prRLN dissection

All patients in this study underwent open surgeries. Neck dissection and thyroid primary tumor resection (ipsilateral lobe + isthmus resection or total thyroidectomy) were performed concurrently. We attempted to subdivide the right cervical central compartment VI into right cervical central VI-1 subzone and VI-2 subzone.

The right cervical central VI-1 subzone dissection of lymph nodes removed all lymphatic and adipose tissues located at layer superficial to the right RLN and around the pretracheal lymph nodes, Delphian lymph nodes, and tracheoesophageal groove lymph nodes. The superior boundary of this dissection was the hyoid bone. The inferior boundary was the suprasternal notch. The external boundary was the inner edge of the common carotid artery, and the inner boundary was the medial line of the trachea.

**Figure 1:**
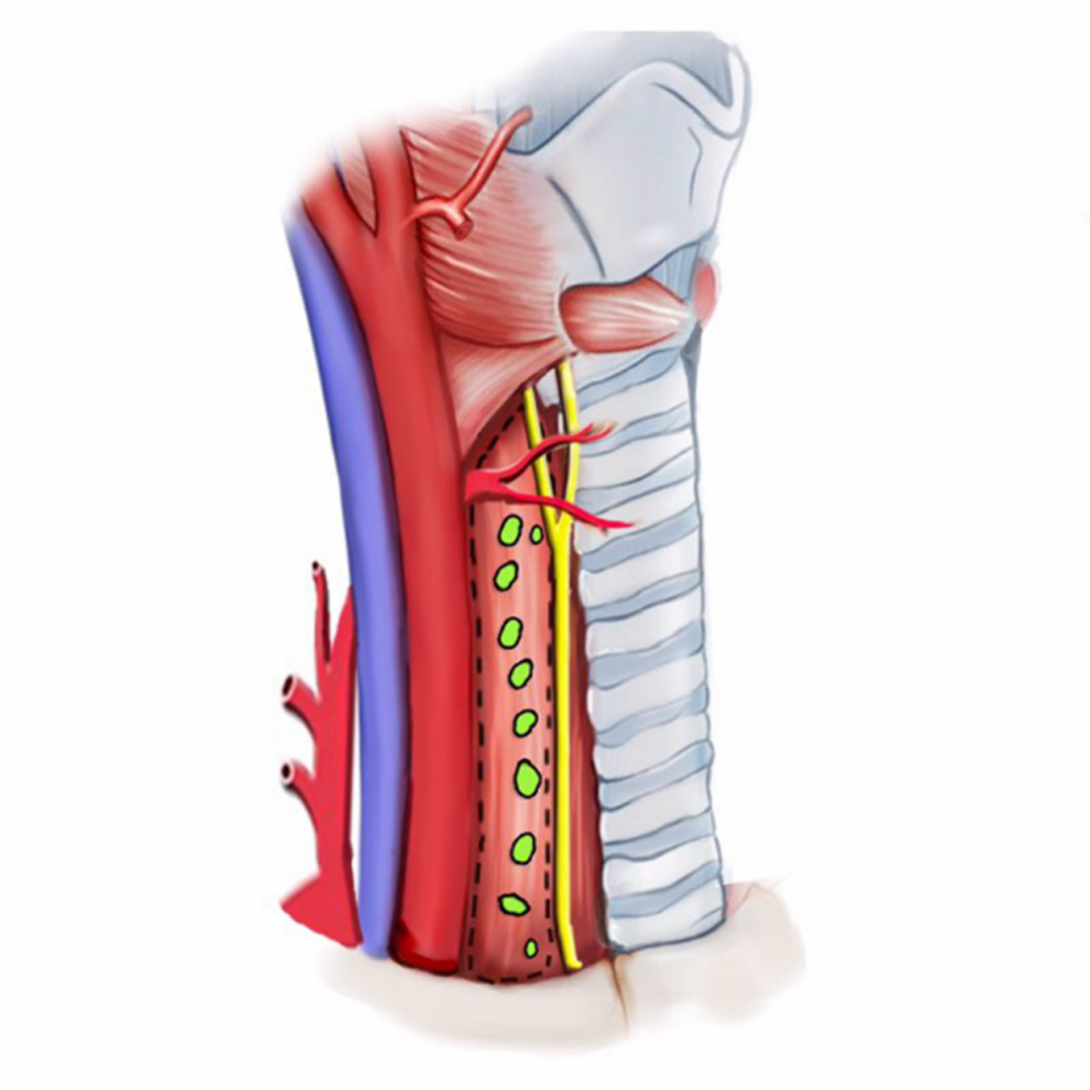
Schematic of right cervical central VI-2 lymph nodes (LN-prRLN)

**Figure 2:**
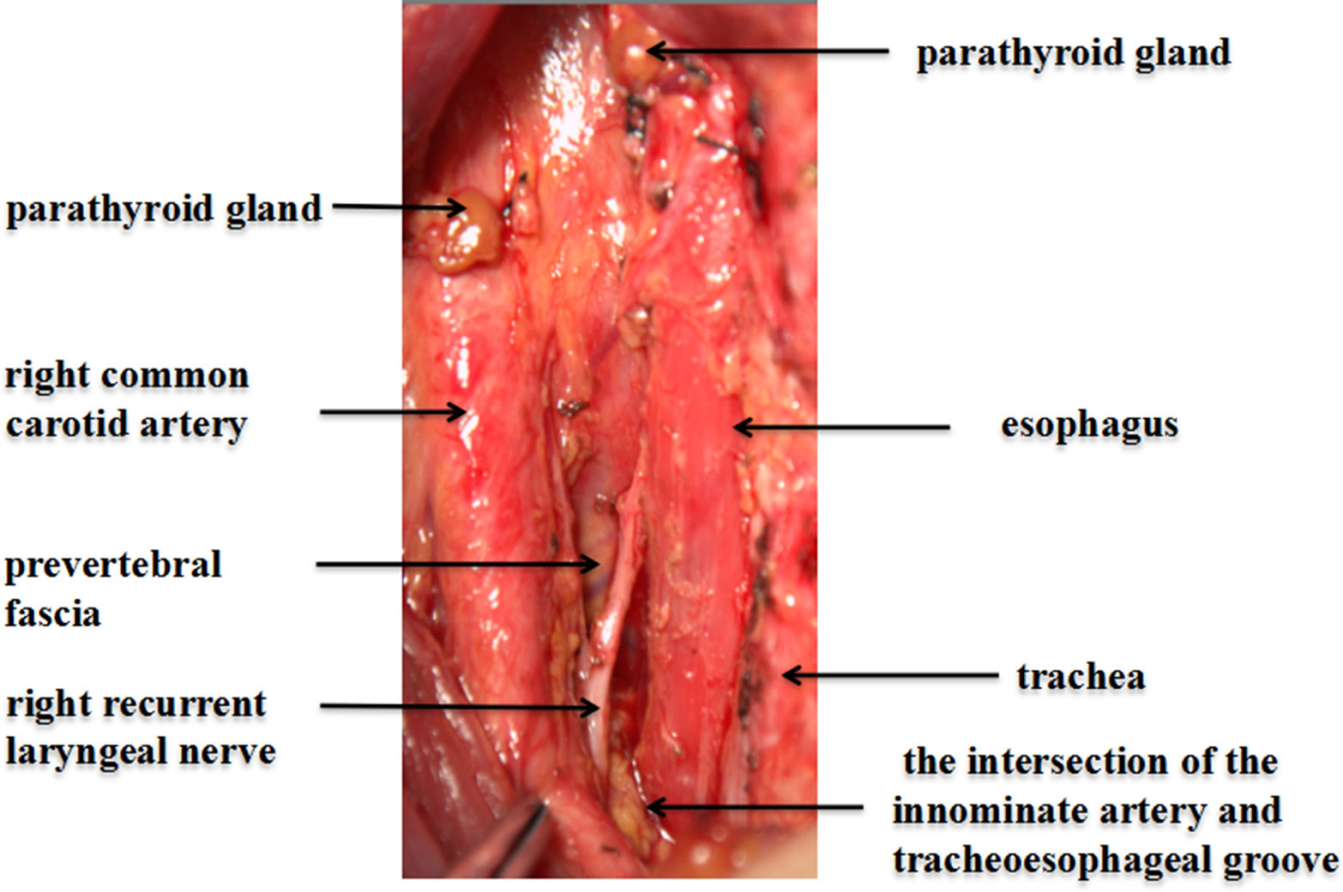
Right cervical central VI-2 after complete lymph node dissection

The right cervical central VI-2 subzone (LN-prRLN) dissection of lymph nodes removed all lymphatic and adipose tissue posterior to the right RLN, in which superior boundary was the laryngeal entry point of the right RLN. The inferior boundary was the intersection of the innominate artery and the tracheoesophageal groove (near the right apical pleura). The inner boundary was the edge of the esophagus; and the external boundary was the inner edge of the common carotid artery. The deepest layer of this dissection reached the deep cervical fascia (prevertebral fascia), and the superficial layer was on the plane of the right RLN (Figures 1 and 2).

All patients in this study underwent a lymph node dissection of both right cervical central VI-1 and VI-2. Additional lateral compartment neck dissections including level II, III, IV and Vb would be performed if metastases were present in this compartment. Level I and Va lymph nodes would be spared unless there were clinically positive lymph nodes.

The cervical RLN was routinely exposed with great care to avoid traction or thermal injury. Its branches were protected as much as possible. The parathyroid was preserved *in situ* if possible or it would be autotransplanted if vascular supply preservation was deemed inadequate. The right apical pleura and blood vessels anterior to the vertebrae were protected. The operating surgeon would mark the lymph node subzones on the excised specimen.

### Pathological examination and v-Raf murine sarcoma viral oncogene homolog B (BRAF) gene mutation testing

Two expert pathologists independently examined the surgical specimens. Specifically, the histological type, number, size, and the presence/ absence of capsular invasion in the primary thyroid tumor were determined. Neck dissection specimens were examined and analysed according to the lymph node subzones. All subzones were carefully explored to locate all lymph nodes. When it was difficult to diagnose lymph node metastasis by routine pathological examination, the immunohistochemistry staining would be employed. The number and size of the metastatic lymph nodes were recorded.

The amplification refractory mutation system (ARMS) was used to detect BRAF gene mutations. This method can detect as low as 1% for the BRAF V600E allele in a wild-type background [16]. The reagent package (Lot number: ADx-FF01, ADx-BR01) was provided by Xiamen Ed biotechnology pharmaceutical Co., Ltd, and the test was carried out in accordance with the manufacturer’s instruction.

### Statistical analysis

Statistical analysis was performed with the aid of the SPSS 20.0 statistical software. The number of harvested lymph nodes was expressed as the median (quartiles) because of its skewed distribution; the differences between groups were assessed using the nonparametric Mann-Whitney test. The chi-squared test or Fisher’s exact test was used for the univariate analysis of the clinical and pathological features and LN-prRLN. A logistic regression analysis was conducted for the multivariate analysis. ROC curves were used to assess the predictive value of potential risk factors. *P* < 0.05 was set as the level of statistical significance.
